# Analysis of four studies in a comparative framework reveals: health linkage consent rates on British cohort studies higher than on UK household panel surveys

**DOI:** 10.1186/1471-2288-14-125

**Published:** 2014-11-27

**Authors:** Gundi Knies, Jonathan Burton

**Affiliations:** ISER University of Essex, Colchester, UK

## Abstract

**Background:**

A number of cohort studies and longitudinal household panel studies in Great Britain have asked for consent to link survey data to administrative health data. We explore commonalities and differences in the process of collecting consent, achieved consent rates and biases in consent with respect to socio-demographic, socio-economic and health characteristics. We hypothesise that British cohort studies which are rooted within the health sciences achieve higher consent rates than the UK household longitudinal studies which are rooted within the social sciences. By contrast, the lack of a specific health focus in household panel studies means there may be less selectivity in consent, in particular, with respect to health characteristics.

**Methods:**

Survey designs and protocols for collecting informed consent to health record linkage on two British cohort studies and two UK household panel studies are systematically compared. Multivariate statistical analysis is then performed on information from one cohort and two household panel studies that share a great deal of the data linkage protocol but vary according to study branding, survey design and study population.

**Results:**

We find that consent is higher in the British cohort studies than in the UK household panel studies, and is higher the more health-focused the study is. There are no systematic patterns of consent bias across the studies and where effects exist within a study or study type they tend to be small. Minority ethnic groups will be underrepresented in record linkage studies on the basis of all three studies.

**Conclusions:**

Systematic analysis of three studies in a comparative framework suggests that the factors associated with consent are idiosyncratic to the study. Analysis of linked health data is needed to establish whether selectivity in consent means the resulting research databases suffer from any biases that ought to be considered.

**Electronic supplementary material:**

The online version of this article (doi:10.1186/1471-2288-14-125) contains supplementary material, which is available to authorized users.

## Background

A number of the UK’s longitudinal surveys^a^ have begun linking their survey population with administrative health records. In the UK, as in many other countries, the survey participants’ informed consent is a necessary pre-requisite in linking survey data with administrative records. There is a growing body of research which suggests that there is some reluctance to consent to data linkage and that consent appears to vary not only with respect to respondent characteristics (see, e.g., [1, 2]) but also with respect to the interview processes and characteristics of the interviewers [3, 4]. Overall, the literature on consent and selectivity in consent, in particular on large-scale social surveys, is as yet very scant and there is little empirical evidence that suggests what level of consent we might expect given the specific study characteristics.

This paper presents empirical results on consent rates and potential consent bias from a systematic comparison of data from two United Kingdom household panel studies and two British birth cohort studies. The research is guided by two hypotheses that emerged from previous research [1]. The first hypothesis is that consent rates to link to health records may be lower in studies that do not have a health focus because the request to participate in a health record linkage study may appear less salient. The second hypothesis is that a study with a specific medical and development focus is more likely to suffer from selection bias into a health record linkage study, leading to increased consent bias in the linked dataset.

To this end we will exploit data from Understanding Society [5], the new UK Household Longitudinal Study (UKHLS), the 1958 National Child Development Study (NCDS) [6] and the 1946 Medical Research Council (MRC) National Survey of Health and Development (NSHD) to replicate and extend previous results reported for the British Household Panel Survey (BHPS) [7], see [1].

## Methods

In this section we will briefly introduce the cohort and household panel studies analysed in the research. The focus will be on outlining the consent procedures and drawing out commonalities and differences in the design. The differences and commonalities will be used to undertake analyses of selectivity in consent, either across all studies or pair-wise.

The research is based solely on secondary analysis of anonymised personal records which are archived and available to researchers using the respective study’s data access route. The research did, therefore, not require formal ethical approval from a research ethics committee.

### Description of the cohort studies

#### The MRC National Survey of Health and Development

The MRC National Survey of Health and Development (NSHD) is a continuing longitudinal birth cohort study consisting of a socially stratified sample of 5,362 (2,547 female and 2,815 male) singleton babies born to married parents in England, Scotland and Wales in a specific week in March 1946. The sample was studied at birth and then a further ten times up to age 15, and then twelve more times in adulthood. The most recent sweep of data collection, at ages 60–64, consisted of a postal questionnaire and then an invitation to visit one of six clinical research facilities across Britain for a health assessment, or to have the more familiar visit at home by a research nurse if they were unable or unwilling to travel. The target sample for the initial postal questionnaire was 3,116 cohort members; of the original sample some had previously refused to take part (n = 669), had emigrated (n = 604), were untraced (n = 337) or had already died (n = 636) [8].

The postal questionnaire collected social and economic information and respondents were asked to report any hospital admissions since last interview. The questionnaire also included a consent form to give the NSHD study team permission to obtain details about the cohort member’s health from their hospital records or their GP (forms available from the NSHD study team on request). To consent to hospital and GP data to be accessed, participants were asked to sign and date the form, and return it by post or facsimile to the MRC Unit. At the time of the survey, the NSHD team had no explicit plans for a comprehensive linkage of survey records with administrative health records, hence there was no information leaflet detailing such plans.

The sample eligible for interview were also invited to participate in a health assessment and an accompanying brochure giving information about the assessments and the risk and benefits of participation accompanied the invitation. The visit took place between two months and two years after the postal questionnaire was sent out.

At the start of the health assessment, a more detailed consent form to participate in the study overall was administered by a research nurse. That consent form did not make any specific references to linkage to administrative health records (materials available from the NSHD study team on request). As part of the administration of the general consent, research nurses were prompted to collect any outstanding consent forms to access hospital and GP records.

The data collection received ethics committee approval and informed consent was obtained for each set of questions and measures. For more detailed information on the general design and implementation of the latest sweep of data collection, see [8].

#### The National Child Development Study

The National Child Development Study (NCDS) is a continuing, longitudinal birth cohort study of 17,415 babies born in Great Britain in one week in 1958. The follow-ups were undertaken when the cohort members were aged 7, 11, 16, 23, 33, 42, 46 and 50 years and, for the first three of these, the birth cohort was also augmented by including immigrants born in the relevant week and identified from school registers in the target sample. At age 50 years, in 2008, 11,461 cohort members were confirmed eligible for interview^b^; of the original sample some had refused to take part (n = 1,337), had emigrated (n = 1,341), were untraced (n = 3,023) or had already died (n = 1,392) [9].

In the same way as the NSHD, the NCDS monitors the cohort member’s health and their physical development, and there was a focus on their educational, social and economic development. Over the years, information has been gathered from a number of sources (e.g.: parents, schools, doctors, medical records, cohort members) and in a variety of ways (e.g.: interview, self-completion, assessments, medical records). The core data collection, however, is through a computer-assisted personal interview (CAPI) which is conducted by a trained interviewer in the cohort member’s home.^c^

In this research we will predominantly draw on information collected in the 8^th^ sweep of data collection, at age 50, which took place in 2008, and when consent was asked. The study protocol, fieldwork materials (including advance letters, information leaflets and consent forms) are included in [9]. The protocol for asking informed consent to link to health records was as follows^d^: Sample members were sent a general advance letter informing them about the next sweep of data collection. Nearer to the interview, there was a second advance letter providing more detailed information on the planned procedures and study content. The study information leaflet (available from the authors on request) mentioned that the interviewer will be asking for the cohort member’s and their partner’s consent to add to the responses provided in the study additional information from routine administrative records held by the National Health Service (NHS). The cohort members were advised that adding the information would only be possible if they provided their consent and that the interviewer would provide them with more information.

Face-to-face interviews with eligible cohort members took place a couple of weeks after the second advance letter had been sent. The consent module was placed at the end of the interview. The interviewer read out the consent preamble, gave an information leaflet on “adding information from routine records” to the respondent and asked them to read it. The respondent could ask the interviewer any further questions. If the respondent was happy to give consent to data linkage, they had to sign and date the consent form and tick the “yes” box next to the health data linkage text, see [10].

The interviewer obtained a copy of the consent form. Consenters also kept a copy for future reference. In addition, the consent outcome was recorded in CAPI.

### Description of the household panel studies

#### The British Household Panel Survey

The British Household Panel Survey (BHPS) is a longitudinal representative sample of the population living in private households in England, Wales and Scotland, in 1991. Additional boost samples for Scotland and Wales were added in 1999 and for Northern Ireland in 2001. The study follows all individuals selected at wave 1 annually and collects information on all members of the household in which the original household members live. The survey collected annual measures on the incidence of serious accidents, the use of health services, health conditions and any long-standing physical or mental impairment, illness or disability. At the 18^th^ sweep of data collection (2008–9), the study asked for consent to link to administrative records held by the NHS and the Department for Work and Pensions (DWP). The study protocol and consent procedures are described in great detail elsewhere [1, 3]. Adult sample members received an advance letter about a week before the interviewer started to make contact with the household. An information leaflet about linking administrative health records was enclosed with the letter. During the interview, interviewers had extra copies of the information leaflet with them. Towards the end of the interview the interviewer gave the respondent a permission form and asked them to sign the form if they were willing to give consent. The consent materials are published as part of the online study documentation, see: https://www.iser.essex.ac.uk/bhps/documentation/pdf_versions/survey_docs/wave18/index.html.

#### Understanding Society: the UK Household Longitudinal Study

The UK Household Longitudinal Study (UKHLS) is a longitudinal representative sample of the population living in private households in the UK in 2009–10. The study follows the lives of around 100,000 people living in around 40,000 households at wave 1. The study incorporates an ethnic minority boost sample, but otherwise follows the design and structure of its predecessor, the BHPS^e^. From the outset, the UKHLS strategy involved the collection of a wide range of health-related information, drawing on standard interviewing and data linkage (implemented from wave 1 onward) as well as the collection of physical health measures, blood samples (implemented in waves 2 and 3) and cognitive measures (implemented in wave 3).

The study asked for consent to link to administrative records held by the NHS at wave 1. Plans to link to administrative records were not mentioned in the study advance letter. Consent was asked at the end of the computer-assisted individual interview, with the interviewer handing over an information leaflet and a consent form for the respondent to sign if they gave permission. The information leaflet about linking administrative records is very similar to the NCDS and BHPS form. It did not, however, include references to GP records and it was not sent with the advance letter. As with the BHPS, the consent materials are made available as part of the online study documentation, see: https://www.understandingsociety.ac.uk/documentation/mainstage/fieldwork-documents.

Table 1 summarizes the main features of the study design and consent procedures of the four studies examined in this research.

**Table 1:** **Overview of commonalities and differences across study samples and consent procedures**

	Cohort studies		Household panel studies	
	NSHD	NCDS	BHPS	UKHLS
***Study team***	MRC Unit for Lifelong Health and Ageing	National Birthday Trust Fund/Centre for Longitudinal Studies	UK Longitudinal Studies Centre	Understanding Society
***Funder***	MRC	National Birthday Trust Fund and ESRC	ESRC	ESRC, Department for Health, and others
***Start year of the study***	1946	1958	1991	2009/2010
***Scope***	GB	GB	GB until 2001, then UK	UK
***Sample description***	Born in a specific week, mainly British White	Born in a specific week, mainly British White	Cross-section of resident population in 1991, additional regional boosts	Cross-section of resident population in 2009/10, additional ethnic minority boost
***Mode of interview***	Postal survey with follow-up home/clinic visit	Face to face interview	Face to face interview	Face to face interview
***Follow-up sequence***	At specific points in time	At specific points in time	Annually	Annually
***Health-related content in survey***	Most	Some	Some	A little^1^
***Number of sweeps before***				
• ***biomarkers collected***	None	None	18^2^	1
• ***consent asked***	22	7	18^2^	None
***Study advance letter includes references to health record linkage***	No^3^	Yes	Yes	No
***Consent asked by***	No-one if postal survey only, else by research nurse	Trained interviewer	Trained interviewer	Trained interviewer
***Information leaflet about linkage***	No^3^	Yes, provided in the interview	Yes, sent in advance	Yes, provided in the interview
***Specific types of health data mentioned***	GP records; Hospital records	Hospital records; GP records; prescriptions	NHS Central Register data; Hospital records; prescriptions; GP records	NHS Central Register data; Hospital records

Notes:

^1^The UKHLS questionnaire collected a large number of subjective well-being indicators and health-related behaviours such as quality of sleep but did not collect information on use of health services as this information would be available through linkage from wave 1 onward.

^2^Consents were first asked in 2008, this was wave 18 for the original BHPS sample, wave 10 for the additional boost samples in Scotland and Wales (which started in 1999) and wave 8 for the additional boost sample in Northern Ireland (which started in 2001).

^3^This is explained by the fact that there were no explicit plans for health record linkage on the NSHD.

### Description of the dependent variable and correlates of consent

Our analysis draws heavily on previous analyses of the BHPS data, see [1]. A small number of changes will apply to the way some of the control variables are derived so as to achieve the greatest level of comparability across the studies. We include socio-demographic and socio-economic characteristics as well as markers of health-related behaviour, reports of diagnoses received and health services used.

On all four studies, our dependent variable is a dichotomous indicator which assumes the value of 1 if the cohort/sample member has returned a signed consent form to the study team which indicates that they agree to the study team accessing their administrative records, and 0 otherwise. On the NSHD the information was coded manually from viewing the consent forms. For the other three studies the information was recorded in CAPI during the face-to-face interview and a consent form had to be signed by the respondent giving consent. All consent forms were also manually checked to ensure that for any consent recorded on the data there was a valid consent form.

## Results

### Survey response rates and consent rates

At the 23rd sweep of data collection on the NSHD, 85 per cent (N = 2,661) of the eligible sample provided information. The majority (N = 2,229) were interviewed and examined at a clinical research facility or in their own homes by research nurses, with others completing only the postal questionnaire (N = 432) [11]. Out of the 2,661 who either had a clinic/home visit or completed a postal questionnaire, 2,505 (94 per cent) agreed to provide consent to GP/hospital records; 141 respondents (5 per cent) did not sign the consent form and 15 (<1 per cent) refused to give their consent. Out of the 2,229 cohort members who either had a clinic/home visit, only 1 person refused to allow access to GP/hospital records, representing a near 100 per cent consent rate. By contrast, among the 432 cohort members who only supplied a postal questionnaire, the consent rate was 64.1 per cent. Seeing as non-consent and non-participation in the health assessment is perfectly collinear, it is not possible to analyse selectivity in consent in our comparative analysis framework. Therefore, the NSHD is dropped from the analyses in this paper.

At the 8^th^ sweep of data collection on the NCDS, 11,461 cohort members were eligible for interview. Of these, 9,758 (85.1 per cent) provided a fully productive interview. All interviewees were asked for consent to link to health records. From them 7,681 (79 per cent) consented to link to health records; of the non-consenters, 1,148 respondents (12 per cent) did not return the consent form and 873 (9 per cent) returned the form but did not give their consent.

At the 18^th^ sweep of data collection on the BHPS, 5,483 households were eligible for interview. Of these, 4,509 (84.2 per cent) participated in the study, providing 11,272 fully productive interviews with adults. All interviewees were asked for consent to link to health records, and 4,568 (40.5 per cent) agreed to data linkage.

The UKHLS started with 55,436 households that were eligible for interview in the 1^st^ sweep of data collection. Of these, 31,346 (56.5 per cent) participated in the study, providing 45,735 fully productive interviews with adults. All interviewees were asked for consent to link to health records, and 32,618 (68.3 per cent) gave consent.

In summary, participation rates (i.e., survey response rates) in the three long-running studies NSHD, NCDS and BHPS were at the same level, amounting to around 85 per cent. At 56.5 per cent the rate was considerable lower in the UKHLS. However, this was the first wave of the UKHLS and it is a common feature of longitudinal samples that participation rates at earlier waves of the study are considerably lower than later on in the life of the panel [12, 13]^f^. For survey methodologists this raises interesting questions over when in the life of the panel it is best to ask for consent [14].

In our classification the NSHD is the most health-focussed, with health and development explicitly part of the title of the study, and funded directly by the MRC. The NCDS is the second most health-focused study, having “child development” in its title and having collected, particularly at the early sweeps, a great deal of medical and health information from the family of the cohort member. The UKHLS and BHPS, funded by the Economic and Social Research Council (ESRC), are ‘branded’ as social science studies and have emphasised the broad range of topics from the beginning of the studies. The UKHLS, of the two household panel surveys, is branded as a “bio-social” survey, with the aim of adding detailed biomarker data to the broad social and economic data collected in the survey. In line with our hypothesis, the overall consent rates in the four studies suggest that the more health-focused cohort studies have higher consent rates than the two general population-based studies. Moreover, within study types it is the more health-focused NSHD (99.9 per cent) that achieved higher consent rate than the less health-focused NCDS (79 per cent), and the same pattern holds for the household panel studies with the more health-focused UKHLS (69.0 per cent) achieving a higher consent rate than the BHPS (41 per cent). Note that we restrict analysis of the UKHLS and BHPS to GB to make a fair comparison between the studies; preliminary results for the UK suggest that consent is much lower in Northern Ireland.

For a description of the samples with respect to all variables used in the analysis, see Additional file 1. Note that we did not include the NSHD in this overview; the data are considered representative of the population aged 60 living in Britain [11], and everybody consented. In other words, there are no health biases in consent to analyse.

### Bivariate associations with consent

For reports of bivariate associations with consent for our independent variables, see Additional file 2. These are split into three blocks, (1) socio-demographic, (2) socio-economic characteristics, and (3) markers of health and related behaviours such as smoking and using health services. Results for the household panel studies consider the complex survey design (which includes oversampling, clustering and stratification) and are calibrated to the population living in Britain in 1991 (BHPS) and 2009 (UKHLS) using the appropriate population weights provided in the studies.

The results suggest that there are some statistically significant associations within all three sets of characteristics in all three studies but there is little evidence for a systematic pattern. This finding itself is interesting since it echoes the general lack of consistent socio-demographic effects found in previous consent research. For example, among the socio-demographic characteristics, only being a member of the British/Irish White population is positively associated with consent in all studies with minority ethnic group members being less likely to consent. Given the larger sample size and the incorporated ethnic minority boost sample, the UKHLS afforded the opportunity to investigate whether the effect is driven by any of the minority ethnic groups in particular. The results suggest that all ethnic groups except White Irish and Mixed groups were less likely to consent than British/Irish White (see Additional file 3). Consent was particularly low among Pakistani and Bangladeshi. This result warrants further investigation in a separate analysis outside our comparative analysis framework.

Results for the household panel studies suggest that those aged 16–24 and those aged 50–52 are more likely to consent. Smaller households, i.e., people living by themselves or with no children have lower consent rates on the NCDS but higher consent rates on the UKHLS. Whilst education is associated with consent at all education levels in the UKHLS except A-Level, there is no association in the NCDS (except no qualifications) and in the BHPS, only those with A-level as their highest qualification are less, and those with or a degree or higher are more, likely to consent; ‘having no qualifications’ is associated with lower consent across all three studies.

A similar (non-)pattern is shown with socio-economic characteristics. Individual gross earnings are associated with consent in the NCDS, with the bottom two quartiles being less likely to consent and the top two quartiles more likely to consent, and there is an association in the bottom quartile (less likely to consent) and third quartile (more likely to consent) on the UKHLS; on the BHPS, there is no association.

Last, but not least, there is only one health-related characteristic that is associated with consent across all three studies, and that is being overweight (or, using our alternative marker, being in the top quartile of the Body Mass Index (BMI)). Having reported a health problem is also positively associated with consent in two studies (NCDS and UKHLS). As to all other characteristics, the associations are either not statistically significant in at least one of the studies, or the direction of the effect differs across studies.

### Multivariate regression analysis

Table 2 reports conventional coefficients from multivariate logistic regression models which include explanatory variables available across the NCDS, BHPS and UKHLS^g^.

**Table 2:** **Logistic regressions on consent to health data linkage: Beta-coefficients**

	NCDS		BHPS		UKHLS	
	Coeff.	S.E.	Coeff.	S.E.	Coeff.	S.E.
England	0.01	0.08	0.22	0.16	-0.11	0.07
London/SE	-0.14*	0.06	0.16	0.11	-0.10*	0.05
Male	-0.02	0.06	0.13*	0.05	0.09***	0.03
British/Irish White	0.33*	0.13	0.74***	0.13	0.47***	0.05
Aged 50-52			0.22	0.14	0.10	0.06
Number of own children in the household (ref: none)						
*1*	-0.10	0.07	-0.02	0.12	0.11*	0.05
*2*	-0.04	0.07	-0.12	0.12	0.08	0.05
*3 or more*	-0.09	0.10	0.24	0.18	0.05	0.08
Lives alone	0.19	0.10	0.12	0.10	-0.13**	0.04
Highest degree (ref: higher degree)						
*First degree*	-0.13	0.16	-0.41*	0.19	0.06	0.06
*Diploma*	-0.19	0.19	-0.56**	0.19	0.15*	0.06
*A-level*	-0.20	0.17	-0.45*	0.21	0.19**	0.07
*Other qualification*	-0.14	0.15	-0.48*	0.19	0.15**	0.05
*No educational qualification*	-0.31	0.16	-0.65**	0.20	-0.02	0.06
Unemployed	0.16	0.18	-0.05	0.22	0.11	0.06
Socio-economic status (ref = managerial/professional)						
*Intermediate*	-0.05	0.10	-0.08	0.11	-0.05	0.06
*Employers*	0.34**	0.11	-0.50*	0.20	-0.11	0.09
*Supervisory*	0.11	0.11	0.05	0.15	0.08	0.07
*Routine*	0.14	0.09	-0.02	0.11	0.11*	0.05
*Other status*	0.20	0.11	-0.13	0.18	-0.09	0.07
*Monthly gross earnings (ref: bottom quartile)*						
*2nd quartile*	0.18*	0.08	-0.10	0.09	0.00	0.04
*3rd quartile*	0.67***	0.09	-0.12	0.19	-0.15*	0.07
*4th quartile*	0.66***	0.09	-0.18	0.19	-0.16*	0.08
Subjective health (ref: excellent)						
*Good*	0.00	0.08	-0.13	0.09	0.02	0.04
*Fair*	-0.28***	0.08	-0.30**	0.11	-0.02	0.05
*Poor*	0.05	0.11	-0.21	0.17	-0.06	0.06
*Very poor*	0.12	0.16	0.04	0.23	-0.02	0.07
Body Mass Index (ref: bottom quartile)						
*2nd quartile*	0.53*	0.25	-0.12	0.19	0.05	0.09
*3rd quartile*	0.54*	0.26	-0.03	0.20	0.01	0.09
*4th quartile*	0.72**	0.26	0.21	0.21	0.12	0.10
Health limits daily activities	-0.03	0.09	0.02	0.12	0.06	0.04
Suffering from an illness	0.17	0.15	0.23	0.16	0.12**	0.05
Reported health problem						
*Diabetes*	-0.16	0.13	0.24	0.13	0.06	0.07
*Relating to stomach problems*	0.21*	0.09	0.05	0.11	-0.04	0.07
*Cancer*	0.11	0.28	0.35	0.25	-0.08	0.13
*Epilepsy*	-0.13	0.28	-0.12	0.31	0.05	0.17
*Relating to chest problems*	0.16	0.08	-0.01	0.10	0.01	0.05
*Other health problem*	-0.00	0.13	0.10	0.18	-0.02	0.04
Constant	0.11	0.34	-0.58	0.38	0.67***	0.14
Number of observations	9,264		5,881		35,536	

Notes:

Significant at ***99%, **95%, *90%.

Results for NCDS not weighted. Results for BHPS and UKHLS weighted and standard errors adjusted for complex survey design.

Source: NCDS Sweep 8, BHPS W18, UKHLS W1.

The results suggest that a number of socio-demographic characteristics are statistically significant factors, in particular in the household panel studies. Those living in London or the South East are less likely to consent (true for both NCDS and UKHLS) and there is a positive association with belonging to the UK White population. The results are inconsistent across studies for the effect of the number of children in the household and whether or not the respondent lives alone – significant only in the UKHLS. If anything, results for the UKHLS suggest that the propensity to consent is higher in multi-person households.

There is some empirical evidence that people’s socio-economic position is associated with the propensity to consent. However, the results go in opposite directions in the different studies. Results on the long-running NCDS and BHPS suggest that those with generally higher levels of education are more likely to consent (note that this is statistically significant only for the BHPS sample) and the opposite is true for the UKHLS sample. Similarly, the association with consent and the respondent’s socio-economic status appears to be idiosyncratic to each study. Last but not least, whilst not being in the bottom quintile of the gross earnings distribution is strongly associated with a higher propensity to consent in the NCDS, the opposite is true for the household panel studies (albeit, this is statistically significant only for the UKHLS).

With respect to markers of health and use of health services, the results suggest that those with fair health are less likely to consent than those with excellent health (true for all studies but statistically significant only for the long-running BHPS and NCDS). Whilst none of the health conditions reported in the household panel studies are associated with the propensity to consent, being in a higher quartile of the BMI and suffering from stomach-related health conditions are associated with a higher propensity to consent on the NCDS.

Regression coefficients in non-linear probability models do not lend themselves to easy interpretation and so we report the corresponding marginal effects in Table 3. The reported marginal effect for, say London/SE, tells us that for two hypothetical individuals with average characteristics, the probability of giving consent is two percentage points lower if the person lives in London/SE than if the person lives elsewhere. Note that some of the effects that were statistically significant in the model reporting beta coefficients may not be statistically significant when expressed as Marginal Effects (ME). This is due to a non-linear transformation of the estimates.

**Table 3:** **Logistic regressions on consent to health data linkage: Marginal effects**

	NCDS		BHPS		UKHLS	
	M.E.	S.E.	M.E.	S.E.	M.E.	S.E.
England	0.00	0.01	0.06	0.04	-0.02	0.01
London/SE	-0.02*	0.01	0.03	0.03	-0.02*	0.01
Male	-0.00	0.01	0.03*	0.01	0.02***	0.00
British/Irish White	0.05*	0.02	0.18***	0.03	0.09***	0.01
Aged 50-52			0.02	0.03	0.02	0.01
Number of own children in the household (ref: none)						
*1*	-0.02	0.01	0.00	0.03	0.02*	0.01
*2*	-0.01	0.01	-0.01	0.03	0.01	0.01
*3 or more*	-0.02	0.02	0.07	0.04	0.01	0.01
Lives alone	0.03	0.02	0.04	0.02	-0.02**	0.01
Highest degree (ref: higher degree)						
*First degree*	-0.02	0.03	-0.08	0.05	0.01	0.01
*Diploma*	-0.03	0.03	-0.12**	0.05	0.03*	0.01
*A-level*	-0.03	0.03	-0.08	0.05	0.03**	0.01
*Other qualification*	-0.02	0.02	-0.10*	0.05	0.03**	0.01
*No educational qualification*	-0.05	0.03	-0.14**	0.05	-0.00	0.01
Unemployed	0.03	0.03	0.02	0.05	0.02	0.01
Socio-economic status (ref = managerial/professional)						
*Intermediate*	-0.01	0.02	-0.01	0.03	-0.01	0.01
*Employers*	0.06**	0.02	-0.11*	0.05	-0.02	0.02
*Supervisory*	0.02	0.02	0.02	0.04	0.02	0.01
*Routine*	0.02	0.01	0.00	0.03	0.02*	0.01
*Other status*	0.03	0.02	-0.03	0.04	-0.02	0.01
*Monthly gross earnings (ref: bottom quartile)*						
*2nd quartile*	0.03*	0.01	-0.02	0.02	0.00	0.01
*3rd quartile*	0.11***	0.01	-0.01	0.05	-0.03*	0.01
*4th quartile*	0.11***	0.01	-0.04	0.05	-0.03*	0.01
Subjective health (ref: excellent)						
*Good*	0.00	0.01	-0.03	0.02	0.00	0.01
*Fair*	-0.04***	0.01	-0.07**	0.03	-0.00	0.01
*Poor*	0.01	0.02	-0.04	0.04	-0.01	0.01
*Very poor*	0.02	0.03	0.01	0.06	-0.00	0.01
Body mass index (ref: bottom quartile)						
*2nd quartile*	0.09*	0.04	-0.05	0.05	0.01	0.02
*3rd quartile*	0.09*	0.04	-0.04	0.05	0.00	0.02
*4th quartile*	0.12**	0.04	0.02	0.05	0.02	0.02
Health limits daily activities	-0.00	0.02	0.00	0.03	0.01	0.01
Suffering from an illness	0.03	0.02	0.05	0.04	0.02**	0.01
Reported health problem						
*Diabetes*	-0.03	0.02	0.05	0.03	0.01	0.01
*Relating to stomach problems*	0.03*	0.02	0.02	0.03	-0.01	0.01
*Cancer*	0.02	0.05	0.10	0.06	-0.02	0.02
*Epilepsy*	-0.02	0.05	-0.01	0.08	0.01	0.03
*Relating to chest problems*	0.03	0.01	0.00	0.02	0.00	0.01
*Other health problem*	-0.00	0.02	-0.01	0.03	-0.00	0.01

Notes:

Significant at ***99%, **95%, *90%.

Results for NCDS not weighted. Results for BHPS and UKHLS weighted and standard errors adjusted for complex survey design.

Source: NCDS Sweep 8, BHPS W18, UKHLS W1.

As can be seen in Table 3, overall the effects are rather small amounting to around 3 percentage points. However, there are a number of greater effects. For instance, if a (hypothetical) person had no educational qualification rather than a higher degree, this would be associated with a 14 percentage point lower probability of consent. Whilst the majority of the larger effects are found on socio-economic and demographic characteristics in the BHPS, the same is true for health related factors in the NCDS. For instance, compared to being in the bottom quartile of the BMI, a person in the top quartile has a 12 percentage point higher probability to consent.

## Discussion

We find, then, that consent rates are higher in the two British birth cohorts which have a more explicit health and development focus than in the general population surveys. When it comes to potential consent bias, we find that there are a higher number of significant socio-demographic factors in the general population surveys and health factors in the birth cohort.

The former finding may be because these general population studies interview adults aged from 16 up, and so cover the whole adult age range, whilst the birth cohort samples are all – by definition – the same age. There is more sample variability, therefore, in the household panel surveys. In addition, the sample sizes of the general population studies were higher than the birth cohorts, particularly the UKHLS which was over 4.5 times the size of the larger birth cohort (NCDS). The larger sample size reduces the standard errors of the estimates, which brings variables into statistical significance which would not be achieved with smaller samples. To alleviate some of these concerns we also estimated the models for a subsample of the UKHLS who were born in the UK and are aged 48–52 (n = 3,144) to make the sample more comparable with the NCDS sample (for results, see Additional file 4), and we estimated the models separately for random fifths of the UKHLS sample (see Additional file 5 for b-coefficients and see Additional file 6 for ME). Both analyses showed that the key results are robust but some effects lack statistical power in smaller samples.

In terms of the socio-economic variables, we find some effect of occupational status in all three studies, although the effect differs across studies with those classified as employers on the NS-SEC 5 being 6 percentage points more likely to consent in the NCDS and 11 percentage points less likely in the BHPS, compared to managerial and professional classes. There is a strong effect of income (monthly gross earnings) in the NCDS, a much weaker effect in UKHLS and no effect in the BHPS.

Measures of health behaviour and health service use did have some strong effects, particularly in the NCDS. The effects are not as strong, though, as those reported in community-based disease studies where it is a typical finding that there are marked biases both in study participation and consent to data linkage for persons who have the studied condition [15]. The NCDS does have a more explicit health focus and study branding and whilst this may increase the consent rate to link to health administrative data (since it is seen as a salient request), it may increase the differences in health between those who are willing to consent and those who withhold it.

There may be other possible explanations for the difference in consent rates between the studies. Whilst we have included in our comparative multivariate analysis all socio-economic and health markers that are available in all three studies (and confirmed the results also across a greater range of variables which were available in any two studies, see endnote number 7), it is possible that less commonly observed health conditions and socio-economic circumstances are more important predictors of consent to health data linkage.

Among the indicators that we have not included in the analysis are markers of survey co-operation, trust and altruism, which some authors have suggested are the strongest correlates of consent, see for example [3, 4, 16]. Whilst these measures may well help explain why, given the composition of the sample, some surveys achieve higher consent rates than others, they are unlikely to bias estimates from health studies using linked data. For completeness, we include estimates also for models including markers of survey co-operation and altruism, see Additional file 7 for b-coefficients and see Additional file 8 for ME. As expected we find that these characteristics are associated with consent to data linkage and this is a consistent finding across all three studies, and in other studies of consent. Also note that associations identified in the main models are robust to inclusion of these measures.

It may be that differences in the survey design (for example the use of annual interviews versus irregular sweeps of data collection, the focus on the cohort member versus focus on all members of the household) explain the difference in the consent rates. We have no means of testing this empirically as this would require experimentation with core elements of the study design. Overall, we feel that design features will not affect consent in a systematic way. For instance, we do not find empirical support for the frequent assertion in the literature that the longer running studies achieve higher consent rates (the BHPS compared to the UKHLS experience would rather suggest the opposite, cf. [14], and the difference in the consent rates between NCDS and UKHLS is not that large). In addition, both the BHPS and UKHLS have annual interviews and the consent rate was very different.

The likely underlying mechanism for the finding that the more health-focused studies achieve higher rates of consent to health data linkage is that the request is more salient to the respondents. The effect may be exacerbated in household panel studies because any one member of the household may view their contribution to the overall study less important than is the case in the cohort studies where about the focus is on the cohort member (although the cohort studies do also interview the cohort member’s partner); and having annual interviews may mean that respondents feel that an additional request is a burden and that they are already giving so much information regularly that linking to administrative data is unnecessary. Again, whilst it will not be possible to change core design features of the study, it is possible to change the design of the consent question so it appears more salient. Experiments have shown, for instance, that consent to (economic) data linkage was higher when the request was made at the start of the interview [17], or in the context of a questionnaire module in the respective domain [14].

## Conclusion

Systematic analysis of three studies in a comparative framework suggests that consent to health record linkage is higher in studies that are more health focused and that the factors associated with consent are idiosyncratic to the study.

Future projects could add data from other surveys such as the English Longitudinal Study of Aging (ELSA), which is a study of those aged 50 and above, or the 1970 British Cohort Study (BCS70) which asked for consent to data linkage at the age 42 survey (2012–2013). In addition, while we have established some consistency across studies in collection of consent to health data linkage, it remains to be seen whether there are differences in match rates and biases with respect to markers of health as recorded in the NHS Central Registers and hospital episodes. All studies examined here are planning to link to the administrative records in the near future.

## Endnotes

^a^This includes a number of studies which are restricted to the area of Great Britain, such as the National Survey of Health and Development (NSHD) and National Child Development Study (NCDS) which are studied here.

^b^This is the number of cohort members who were confirmed eligible for interview during the 2008 fieldwork; a total of 12,316 cases were issued to field. Reported numbers of ineligible cases consider known ineligibility before issued to field and additional cases confirmed ineligible during fieldwork as per tables 2.2 and 6.1 in [9].

^c^Although the age 46 survey was by telephone and the age 55 survey is sequential mixed mode - web then telephone.

^d^Shepherd [10] provides a general overview of procedures for informed consent on the NCDS, including consenting to participate in the study.

^e^The BHPS sample was incorporated into the UKHLS from Wave 2.

^f^After refusal to the original survey and then drop out at the next couple of waves, in the later stages of panel studies those people who are more likely to refuse to participate will have already dropped out, and so the people remaining in the sample tend to be the more co-operative members of the original sample, and so response rates then become higher.

^g^For the long-running BHPS and NCDS it was also possible to include a longer list of health conditions and to compare associations with use of health services (results not reported). This showed that there is no association with conditions related to sight, hearing, allergies and migraine in either of the studies. The same is true for use of health services in the last year (i.e., GP practice, out-patient and inpatient hospital stays). There was a negative association with having private health insurance but this was statistically significant only in the NCDS study.

## Electronic supplementary material

Additional file 1: Table S1: Summary statistics. Summary statistics of all variables used in the research for the NCDS, BHPS and UKHLS studies. (DOCX 38 KB)

Additional file 2: Table S2: Population estimates of bivariate association with consent. Bivariate associations with consent of all variables used in the research for the NCDS, BHPS and UKHLS studies. (DOCX 33 KB)

Additional file 3: Table S3: Logistic regressions on consent to health data linkage considering detailed ethnic categories using the UKHLS. Beta coefficients and marginal effects. Logistic regressions on consent to health data linkage for the UKHLS; includes a detailed self-reported ethnic group classification. (DOCX 24 KB)

Additional file 4: Table S4: Logistic regressions on consent to health data linkage on UKHLS sample born in the UK and aged 48–52 (N = 3,144). Beta coefficients and marginal effects. Logistic regressions on consent to health data linkage for the UKHLS; focusing on a subsample of the population that is similar in age to the population studied in the NCDS. (DOCX 18 KB)

Additional file 5: Table S5: Logistic regressions on consent to health data linkage on five random samples (RS) of the UKHLS. Beta coefficients. Logistic regressions on consent to health data linkage for the UKHLS; focusing on five random subsamples of the population so that the sample size is more similar to that studied in the NCDS and the BHPS. Results reported as beta coefficients. (DOCX 23 KB)

Additional file 6: Table S6: Logistic regressions on consent to health data linkage on five random samples (RS) of the UKHLS. Marginal effects. Logistic regressions on consent to health data linkage for the UKHLS; focusing on five random subsamples of the population so that the sample size is more similar to that studied in the NCDS and the BHPS. Results reported as marginal effects. (DOCX 22 KB)

Additional file 7: Table S7: Logistic regressions on consent to health data linkage including markers of survey co-operation. Beta coefficients. Logistic regressions on consent to health data linkage for the NCDS, BHPS and UKHLS studies; includes additional predictor variables to allow for heterogeneity in the study participants’ degree of survey co-operation. Results reported as beta co-efficients. (DOCX 21 KB)

Additional file 8: Table S8: Logistic regressions on consent to health data linkage including markers of survey co-operation. Marginal effects. Logistic regressions on consent to health data linkage for the NCDS, BHPS and UKHLS studies; includes additional predictor variables to allow for heterogeneity in the study participants’ degree of survey co-operation. Results reported as marginal effects. (DOCX 21 KB)
