# Supplementary material for: Analysis of four studies in a comparative framework reveals: health linkage consent rates on British cohort studies higher than on UK household panel surveys
Source: BMC Med Res Methodol. 2014 Nov 27;14:125. doi: 10.1186/1471-2288-14-125 (PMC4280701; doi:10.1186/1471-2288-14-125)
Supplement: Supplementary file 1 — Additional file 1: Table S1: Summary statistics. Summary statistics of all variables used in the research for the NCDS, BHPS and UKHLS studies. (DOCX 38 KB) [file 12874_2014_1141_MOESM1_ESM.docx]

**Table S1 - Summary statistics**

|  | **NCDS** | | | | | | **BHPS** | | | | | | | **UKHLS** | | | | | |
| --- | --- | --- | --- | --- | --- | --- | --- | --- | --- | --- | --- | --- | --- | --- | --- | --- | --- | --- | --- |
|  | Mean^1^ | S.D. | Min | Max | N | Mean^1^ | | S.D. | Min | | Max | | N | Mean^1^ | S.D. | Min | Max | N |  |
| Consent to health data linkage | 0.79 | 0.41 | 0 | 1 | 9,740 | 0.41 | | 0.49 | 0 | | 1 | | 11,272 | 0.69 | 0.46 | 0 | 1 | 45,735 |  |
| Country of residence |  |  |  |  |  |  | |  |  | |  | |  |  |  |  |  |  |  |
| *England* | 0.85 | 0.36 | 0 | 1 | 9,740 | 0.59 | | 0.49 | 0 | | 1 | | 11,272 | 0.88 | 0.33 | 0 | 1 | 45,735 |  |
| *Wales* | 0.10 | 0.29 | 0 | 1 | 9,740 | 0.21 | | 0.41 | 0 | | 1 | | 11,272 | 0.05 | 0.22 | 0 | 1 | 45,735 |  |
| *Scotland* | 0.06 | 0.23 | 0 | 1 | 9,740 | 0.20 | | 0.40 | 0 | | 1 | | 11,272 | 0.07 | 0.26 | 0 | 1 | 45,735 |  |
| London/Southeast | 0.23 | 0.42 | 0 | 1 | 9,740 | 0.15 | | 0.35 | 0 | | 1 | | 11,221 | 0.29 | 0.46 | 0 | 1 | 45,735 |  |
| Male | 0.49 | 0.50 | 0 | 1 | 9,740 | 0.45 | | 0.50 | 0 | | 1 | | 11,272 | 0.44 | 0.50 | 0 | 1 | 45,735 |  |
| British/Irish White | 0.96 | 0.19 | 0 | 1 | 9,739 | 0.94 | | 0.24 | 0 | | 1 | | 10,935 | 0.76 | 0.43 | 0 | 1 | 45,682 |  |
| Age |  |  |  |  |  | 46.7 | | 19.0 | 15 | | 99 | | 11,271 | 45.9 | 18.1 | 16 | 101 | 45,735 |  |
| Aged 50-52 |  |  |  |  |  | 0.05 | | 0.21 | 0 | | 1 | | 11,272 | 0.05 | 0.22 | 0 | 1 | 45,735 |  |
| Number of own children in the household | 1.02 | 1.04 | 0 | 9 | 9,718 | 0.45 | | 0.83 | 0 | | 3 | | 11,272 | 0.53 | 0.96 | 0 | 10 | 45,735 |  |
| Lives alone | 0.11 | 0.31 | 0 | 1 | 9,740 | 0.14 | | 0.35 | 0 | | 1 | | 11,272 | 0.16 | 0.36 | 0 | 1 | 45,735 |  |
| Highest level of education |  |  |  |  |  |  | |  |  | |  | |  |  |  |  |  |  |  |
| *higher degree* | 0.04 | 0.19 | 0 | 1 | 9,740 | 0.03 | | 0.17 | 0 | | 1 | | 11,059 | 0.09 | 0.28 | 0 | 1 | 45,695 |  |
| *degree* | 0.16 | 0.37 | 0 | 1 | 9,740 | 0.13 | | 0.33 | 0 | | 1 | | 11,059 | 0.13 | 0.34 | 0 | 1 | 45,695 |  |
| *diploma* | 0.05 | 0.21 | 0 | 1 | 9,740 | 0.33 | | 0.47 | 0 | | 1 | | 11,059 | 0.10 | 0.30 | 0 | 1 | 45,695 |  |
| *a-level* | 0.09 | 0.28 | 0 | 1 | 9,740 | 0.13 | | 0.34 | 0 | | 1 | | 11,059 | 0.08 | 0.27 | 0 | 1 | 45,695 |  |
| *other qualification* | 0.48 | 0.50 | 0 | 1 | 9,740 | 0.22 | | 0.42 | 0 | | 1 | | 11,059 | 0.39 | 0.49 | 0 | 1 | 45,695 |  |
| *no qualification* | 0.19 | 0.39 | 0 | 1 | 9,740 | 0.16 | | 0.37 | | 0 | 1 | | 11,059 | 0.17 | 0.38 | 0 | 1 | 45,695 |  |
| Unemployed | 0.03 | 0.16 | 0 | 1 | 9,740 | 0.03 | | 0.18 | | 0 | 1 | | 11,271 | 0.07 | 0.25 | 0 | 1 | 45,733 |  |
| Socio-economic group |  |  |  |  |  |  | |  | |  |  | |  |  |  |  |  |  |  |
| *manager* | 0.39 | 0.49 | 0 | 1 | 9,714 | 0.24 | | 0.43 | | 0 | 1 | | 11,183 | 0.22 | 0.42 | 0 | 1 | 45,574 |  |
| *intermediate* | 0.10 | 0.30 | 0 | 1 | 9,714 | 0.09 | | 0.28 | | 0 | 1 | | 11,183 | 0.08 | 0.27 | 0 | 1 | 45,574 |  |
| *employers* | 0.10 | 0.30 | 0 | 1 | 9,714 | 0.05 | | 0.22 | | 0 | 1 | | 11,183 | 0.05 | 0.22 | 0 | 1 | 45,574 |  |
| *supervisory* | 0.09 | 0.28 | 0 | 1 | 9,714 | 0.05 | | 0.22 | | 0 | 1 | | 11,183 | 0.04 | 0.20 | 0 | 1 | 45,574 |  |
| *routine* | 0.17 | 0.37 | 0 | 1 | 9,714 | 0.17 | | 0.38 | | 0 | 1 | | 11,183 | 0.16 | 0.36 | 0 | 1 | 45,574 |  |
| *other status* | 0.15 | 0.36 | 0 | 1 | 9,714 | 0.39 | | 0.49 | | 0 | 1 | | 11,183 | 0.45 | 0.50 | 0 | 1 | 45,574 |  |
| Mean gross earnings (monthly, in £1k) | 1.59 | 4.65 | 0 | 304 | 9,740 | 0.96 | | 1.33 | | 0 | | 24.0 | 11,272 | 0.88 | 1.63 | 0 | 159 | 45,735 |  |
| Mean household income (monthly, in £1k) |  |  |  |  |  | 3.30 | | 2.67 | | 0 | | 86.7 | 11,272 | 3.31 | 3.39 | 0 | 166 | 45,677 |  |
| Supports left-wing party | 0.54 | 0.50 | 0 | 1 | 9,740 | 0.41 | | 0.49 | | 0 | | 1 | 11,272 | 0.30 | 0.46 | 0 | 1 | 45,735 |  |
| Non-voter | 0.10 | 0.29 | 0 | 1 | 9,740 | 0.16 | | 0.37 | | 0 | | 1 | 11,272 | 0.04 | 0.19 | 0 | 1 | 45,735 |  |
| Refused question on income | 0.02 | 0.15 | 0 | 1 | 9,740 | 0.02 | | 0.13 | | 0 | | 1 | 11,272 | 0.04 | 0.19 | 0 | 1 | 45,735 |  |
| Generally trusts others | 0.53 | 0.50 | 0 | 1 | 9,728 | 0.32 | | 0.47 | | 0 | | 1 | 11,075 | 0.34 | 0.47 | 0 | 1 | 37,999 |  |
| Subjective health |  |  |  |  |  |  | |  | |  | |  |  |  |  |  |  |  |  |
| *excellent* | 0.19 | 0.40 | 0 | 1 | 9,731 | 0.22 | | 0.41 | | 0 | | 1 | 11,266 | 0.18 | 0.38 | 0 | 1 | 45,658 |  |
| *good* | 0.33 | 0.47 | 0 | 1 | 9,731 | 0.48 | | 0.50 | | 0 | | 1 | 11,266 | 0.32 | 0.47 | 0 | 1 | 45,658 |  |
| *fair* | 0.29 | 0.45 | 0 | 1 | 9,731 | 0.21 | | 0.41 | | 0 | | 1 | 11,266 | 0.28 | 0.45 | 0 | 1 | 45,658 |  |
| *poor* | 0.13 | 0.33 | 0 | 1 | 9,731 | 0.07 | | 0.26 | | 0 | | 1 | 11,266 | 0.15 | 0.35 | 0 | 1 | 45,658 |  |
| *very poor* | 0.06 | 0.23 | 0 | 1 | 9,731 | 0.02 | | 0.13 | | 0 | | 1 | 11,266 | 0.07 | 0.26 | 0 | 1 | 45,658 |  |
| Current smoker | 0.19 | 0.40 | 0 | 1 | 9,740 | 0.22 | | 0.41 | | 0 | | 1 | 11,272 | n.a. |  |  |  |  |  |
| Smokes 20 or more cigarettes daily | 0.03 | 0.16 | 0 | 1 | 9,740 | 0.02 | | 0.15 | | 0 | | 1 | 11,272 | n.a. |  |  |  |  |  |
| Body Mass Index (categories) |  |  |  |  |  |  | |  | |  | |  |  |  |  |  |  |  |  |
| *Underweight* | 0.01 | 0.09 | 0 | 1 | 9,329 | 0.02 | | 0.14 | | 0 | | 1 | 9,363 | 0.02 | 0.15 | 0 | 1 | 42,605 |  |
| *Normal weight* | 0.36 | 0.48 | 0 | 1 | 9,329 | 0.45 | | 0.50 | | 0 | | 1 | 9,363 | 0.44 | 0.50 | 0 | 1 | 42,605 |  |
| *Overweight* | 0.39 | 0.49 | 0 | 1 | 9,329 | 0.36 | | 0.48 | | 0 | | 1 | 9,363 | 0.35 | 0.48 | 0 | 1 | 42,605 |  |
| *Obese* | 0.24 | 0.43 | 0 | 1 | 9,329 | 0.17 | | 0.37 | | 0 | | 1 | 9,363 | 0.18 | 0.39 | 0 | 1 | 42,605 |  |
| (Registered) disabled | 0.04 | 0.20 | 0 | 1 | 9,728 | 0.10 | | 0.30 | | 0 | | 1 | 11,272 | n.a. |  |  |  |  |  |
| Has reported limiting health problem | 0.16 | 0.36 | 0 | 1 | 9,740 | 0.17 | | 0.37 | | 0 | | 1 | 11,272 | 0.29 | 0.45 | 0 | 1 | 45,735 |  |
| Has reported any health problem | 0.89 | 0.31 | 0 | 1 | 9,732 | 0.60 | | 0.49 | | 0 | | 1 | 11,272 | 0.28 | 0.45 | 0 | 1 | 45,735 |  |
| Suffering from listed health problem |  |  |  |  |  |  | |  | |  | |  |  |  |  |  |  |  |  |
| *diabetes* | 0.04 | 0.20 | 0 | 1 | 9,732 | 0.05 | | 0.22 | | 0 | | 1 | 11,272 | 0.06 | 0.23 | 0 | 1 | 45,735 |  |
| *stomach* | 0.10 | 0.30 | 0 | 1 | 9,732 | 0.08 | | 0.27 | | 0 | | 1 | 11,272 | 0.04 | 0.19 | 0 | 1 | 45,735 |  |
| *cancer* | 0.01 | 0.10 | 0 | 1 | 9,732 | 0.02 | | 0.13 | | 0 | | 1 | 11,272 | 0.01 | 0.10 | 0 | 1 | 45,735 |  |
| *epilepsy* | 0.01 | 0.09 | 0 | 1 | 9,732 | 0.01 | | 0.09 | | 0 | | 1 | 11,272 | 0.01 | 0.09 | 0 | 1 | 45,735 |  |
| *chest* | 0.13 | 0.33 | 0 | 1 | 9,732 | 0.14 | | 0.35 | | 0 | | 1 | 11,272 | 0.11 | 0.31 | 0 | 1 | 45,735 |  |
| *sight* | 0.67 | 0.47 | 0 | 1 | 9,732 | 0.06 | | 0.23 | | 0 | | 1 | 11,272 | n.a. |  |  |  |  |  |
| *hearing* | 0.10 | 0.30 | 0 | 1 | 9,732 | 0.09 | | 0.29 | | 0 | | 1 | 11,272 | n.a. |  |  |  |  |  |
| *allergy* | 0.20 | 0.40 | 0 | 1 | 9,732 | 0.13 | | 0.33 | | 0 | | 1 | 11,272 | n.a. |  |  |  |  |  |
| *migraine* | 0.08 | 0.28 | 0 | 1 | 9,732 | 0.07 | | 0.26 | | 0 | | 1 | 11,272 | n.a. |  |  |  |  |  |
| *cardio vascular* | n.a. |  |  |  |  | 0.19 | | 0.40 | | 0 | | 1 | 11,272 | 0.16 | 0.37 | 0 | 1 | 45,735 |  |
| *other health problem* | 0.84 | 0.36 | 0 | 1 | 9,740 | 0.54 | | 0.50 | | 0 | | 1 | 11,272 | 0.43 | 0.50 | 0 | 1 | 45,735 |  |
| Privately insured | 0.27 | 0.44 | 0 | 1 | 9,740 | 0.15 | | 0.35 | | 0 | | 1 | 11,272 | n.a. |  |  |  |  |  |
| Hospital stay (last 12 months) | 0.28 | 0.45 | 0 | 1 | 9,733 | 0.25 | | 0.43 | | 0 | | 1 | 11,272 | n.a. |  |  |  |  |  |
| Hospital out-patient (last 12 months) | 0.56 | 0.50 | 0 | 1 | 9,740 | 0.17 | | 0.38 | | 0 | | 1 | 11,272 | n.a. |  |  |  |  |  |
| Reported to have seen a doctor (last 12 months) | 0.68 | 0.47 | 0 | 1 | 9,740 | 0.76 | | 0.43 | | 0 | | 1 | 11,272 | n.a. |  |  |  |  |  |

Figures are based on raw frequencies; for the household panel studies the characteristics will not be representative given the complex survey design.

^1^ This is the proportion of the population in case of dichotomous variables.

Source: NCDS Sweep 8, BHPS W18, UKHLS W1.
