# Supplementary material for: Analysis of four studies in a comparative framework reveals: health linkage consent rates on British cohort studies higher than on UK household panel surveys
Source: BMC Med Res Methodol. 2014 Nov 27;14:125. doi: 10.1186/1471-2288-14-125 (PMC4280701; doi:10.1186/1471-2288-14-125)
Supplement: Supplementary file 2 — Additional file 2: Table S2: Population estimates of bivariate association with consent. Bivariate associations with consent of all variables used in the research for the NCDS, BHPS and UKHLS studies. (DOCX 33 KB) [file 12874_2014_1141_MOESM2_ESM.docx]

**Table S2 - Population estimates of bivariate association with consent.**

| **Population characteristic** | **NCDS** | | | **BHPS** | | | **UKHLS** | | |
| --- | --- | --- | --- | --- | --- | --- | --- | --- | --- |
|  | *Consent rate* | *S.E.* | *p-value^1^* | *Consent rate* | *S.E.* | *p-value^1^* | *Consent rate* | *S.E.* | *p-value^1^* |
| Total population (baseline) | 0.790 | 0.00 |  | 0.410 | 0.01 |  | 0.706 | 0.004 |  |
| Country of residence |  |  |  |  |  |  |  |  |  |
| *England* | 0.790 | 0.00 | 0.81 | 0.420 | 0.01 | 0.05 | 0.700 | 0.00 | 0.00 |
| *Wales* | 0.800 | 0.01 | 0.25 | 0.320 | 0.05 | 0.04 | 0.730 | 0.02 | 0.18 |
| *Scotland* | 0.770 | 0.02 | 0.28 | 0.380 | 0.05 | 0.45 | 0.740 | 0.01 | 0.00 |
| London/South East | 0.770 | 0.01 | 0.01 | 0.450 | 0.02 | 0.05 | 0.660 | 0.01 | 0.00 |
| Male | 0.800 | 0.01 | 0.05 | 0.420 | 0.01 | 0.35 | 0.710 | 0.00 | 0.00 |
| British/Irish White | 0.790 | 0.00 | 0.00 | 0.430 | 0.01 | 0.00 | 0.720 | 0.00 | 0.00 |
| Age group |  |  |  |  |  |  |  |  |  |
| *aged 16-24* | n.a. |  |  | 0.460 | 0.02 | 0.01 | 0.750 | 0.01 | 0.00 |
| *aged 25-39* | n.a. |  |  | 0.430 | 0.02 | 0.28 | 0.700 | 0.01 | 0.04 |
| *aged 40-49* | n.a. |  |  | 0.400 | 0.02 | 0.28 | 0.700 | 0.01 | 0.62 |
| *aged 50-59* | n.a. |  |  | 0.400 | 0.02 | 0.22 | 0.710 | 0.01 | 0.33 |
| *aged 50-52* | See total |  |  | 0.420 | 0.03 | 0.69 | 0.730 | 0.01 | 0.04 |
| *aged 60 or older* | n.a. |  |  | 0.400 | 0.02 | 0.28 | 0.690 | 0.01 | 0.00 |
| Number of own children in the household |  |  |  |  |  |  |  |  |  |
| *no children* | 0.800 | 0.01 | 0.02 | 0.420 | 0.012 | 0.662 | 0.700 | 0.00 | 0.01 |
| *one child* | 0.780 | 0.01 | 0.21 | 0.410 | 0.024 | 0.993 | 0.720 | 0.01 | 0.01 |
| *two children* | 0.790 | 0.01 | 0.66 | 0.390 | 0.024 | 0.210 | 0.710 | 0.01 | 0.26 |
| *three or more children* | 0.770 | 0.01 | 0.16 | 0.450 | 0.040 | 0.313 | 0.700 | 0.01 | 0.60 |
| Lives alone | 0.820 | 0.01 | 0.01 | 0.420 | 0.020 | 0.617 | 0.670 | 0.01 | 0.00 |
| Highest level of education |  |  |  |  |  |  |  |  |  |
| *higher degree* | 0.800 | 0.02 | 0.49 | 0.500 | 0.04 | 0.03 | 0.660 | 0.01 | 0.00 |
| *Degree* | 0.800 | 0.01 | 0.35 | 0.420 | 0.02 | 0.64 | 0.690 | 0.01 | 0.02 |
| *Diploma* | 0.780 | 0.02 | 0.68 | 0.410 | 0.01 | 0.46 | 0.720 | 0.01 | 0.02 |
| *a-level* | 0.780 | 0.01 | 0.40 | 0.460 | 0.02 | 0.01 | 0.720 | 0.01 | 0.19 |
| *other qualification* | 0.800 | 0.01 | 0.12 | 0.420 | 0.02 | 0.67 | 0.730 | 0.00 | 0.00 |
| *no qualification* | 0.770 | 0.01 | 0.02 | 0.360 | 0.02 | 0.00 | 0.660 | 0.01 | 0.00 |
| Unemployed | 0.780 | 0.03 | 0.73 | 0.410 | 0.04 | 0.86 | 0.720 | 0.01 | 0.12 |
| Socio-economic group |  |  |  |  |  |  |  |  |  |
| *Manager* | 0.800 | 0.01 | 0.11 | 0.430 | 0.01 | 0.09 | 0.710 | 0.01 | 0.79 |
| *Intermediate* | 0.780 | 0.01 | 0.71 | 0.410 | 0.02 | 0.69 | 0.710 | 0.01 | 0.90 |
| *Employers* | 0.770 | 0.01 | 0.09 | 0.360 | 0.03 | 0.03 | 0.710 | 0.01 | 0.53 |
| *Supervisory* | 0.810 | 0.01 | 0.08 | 0.450 | 0.03 | 0.30 | 0.720 | 0.01 | 0.15 |
| *Routine* | 0.800 | 0.01 | 0.34 | 0.430 | 0.02 | 0.37 | 0.730 | 0.01 | 0.00 |
| *other status* | 0.760 | 0.01 | 0.00 | 0.400 | 0.01 | 0.14 | 0.700 | 0.00 | 0.00 |
| Quartile of monthly gross earnings |  |  |  |  |  |  |  |  |  |
| *bottom quartile* | 0.730 | 0.01 | 0.00 | 0.410 | 0.02 | 0.70 | 0.700 | 0.01 | 0.09 |
| *2nd quartile* | 0.770 | 0.01 | 0.00 | 0.400 | 0.02 | 0.13 | 0.700 | 0.01 | 0.56 |
| *3rd quartile* | 0.830 | 0.01 | 0.00 | 0.430 | 0.02 | 0.28 | 0.720 | 0.01 | 0.02 |
| *top quartile* | 0.820 | 0.01 | 0.00 | 0.420 | 0.01 | 0.39 | 0.710 | 0.01 | 0.94 |
| Quartile of household income |  |  |  |  |  |  |  |  |  |
| *bottom quartile* | n.a. |  |  | 0.410 | 0.02 | 0.60 | 0.670 | 0.01 | 0.00 |
| *2nd quartile* | n.a. |  |  | 0.400 | 0.02 | 0.38 | 0.710 | 0.01 | 0.08 |
| *3rd quartile* | n.a. |  |  | 0.450 | 0.02 | 0.01 | 0.720 | 0.01 | 0.00 |
| *top quartile* | n.a. |  |  | 0.390 | 0.02 | 0.17 | 0.710 | 0.01 | 0.21 |
| Subjective health |  |  |  |  |  |  |  |  |  |
| *Excellent* | 0.800 | 0.01 | 0.47 | 0.440 | 0.02 | 0.03 | 0.690 | 0.01 | 0.03 |
| *Good* | 0.800 | 0.01 | 0.06 | 0.420 | 0.01 | 0.71 | 0.710 | 0.01 | 0.08 |
| *Fair* | 0.760 | 0.01 | 0.00 | 0.380 | 0.02 | 0.02 | 0.710 | 0.01 | 0.64 |
| *Poor* | 0.810 | 0.01 | 0.06 | 0.400 | 0.03 | 0.63 | 0.700 | 0.01 | 0.51 |
| *very poor* | 0.800 | 0.02 | 0.43 | 0.430 | 0.05 | 0.76 | 0.710 | 0.01 | 0.82 |
| Current smoker | 0.800 | 0.01 | 0.12 | 0.420 | 0.02 | 0.71 | n.a. |  |  |
| Smokes 20 or more cigarettes daily | 0.830 | 0.02 | 0.13 | 0.430 | 0.04 | 0.76 | n.a. |  |  |
| Body Mass Index (categories) |  |  |  |  |  |  |  |  |  |
| *Underweight* | 0.690 | 0.05 | 0.02 | 0.460 | 0.05 | 0.33 | 0.690 | 0.02 | 0.14 |
| *Normal weight* | 0.790 | 0.01 | 0.27 | 0.400 | 0.01 | 0.04 | 0.710 | 0.00 | 0.04 |
| *Overweight* | 0.790 | 0.01 | 0.34 | 0.410 | 0.02 | 0.62 | 0.710 | 0.00 | 0.54 |
| *Obese* | 0.810 | 0.01 | 0.01 | 0.460 | 0.02 | 0.00 | 0.730 | 0.01 | 0.00 |
| (Registered) disabled | 0.790 | 0.02 | 0.92 | 0.390 | 0.02 | 0.19 | 0.700 | 0.00 | 0.73 |
| Has reported limiting health problem | 0.790 | 0.01 | 0.99 | 0.390 | 0.02 | 0.23 | 0.700 | 0.01 | 0.71 |
| Has reported any health problem | 0.790 | 0.00 | 0.03 | 0.420 | 0.01 | 0.26 | 0.720 | 0.01 | 0.01 |
| Suffering from listed health problem | 0.780 | 0.02 | 0.74 | 0.460 | 0.03 | 0.06 | 0.710 | 0.01 | 0.63 |
| *diabetes* | 0.820 | 0.01 | 0.01 | 0.430 | 0.02 | 0.40 | 0.700 | 0.01 | 0.60 |
| *stomach* | 0.810 | 0.04 | 0.64 | 0.480 | 0.06 | 0.21 | 0.720 | 0.02 | 0.63 |
| *cancer* | 0.740 | 0.05 | 0.25 | 0.400 | 0.07 | 0.86 | 0.730 | 0.03 | 0.45 |
| *epilepsy* | 0.810 | 0.01 | 0.03 | 0.410 | 0.02 | 0.98 | 0.720 | 0.01 | 0.02 |
| *chest* | 0.800 | 0.01 | 0.47 | 0.440 | 0.02 | 0.03 | 0.690 | 0.01 | 0.03 |
| Has listed health problem |  |  |  |  |  |  |  |  |  |
| *sight* | 0.790 | 0.01 | 0.10 | 0.420 | 0.03 | 0.88 | n.a. |  |  |
| *hearing* | 0.800 | 0.01 | 0.39 | 0.440 | 0.02 | 0.20 | n.a. |  |  |
| *allergy* | 0.800 | 0.01 | 0.39 | 0.450 | 0.02 | 0.03 | n.a. |  |  |
| *migraine* | 0.800 | 0.01 | 0.49 | 0.470 | 0.03 | 0.02 | n.a. |  |  |
| *cardio vascular* | n.a. | n.a. | n.a. | 0.400 | 0.02 | 0.43 | 0.710 | 0.01 | 0.29 |
| *other health problem* | 0.790 | 0.00 | 0.04 | 0.420 | 0.01 | 0.52 | 0.710 | 0.00 | 0.01 |
| Privately insured | 0.760 | 0.01 | 0.00 | 0.430 | 0.02 | 0.51 | n.a. |  |  |
| Hospital stay (last 12 months) | 0.790 | 0.01 | 0.52 | 0.430 | 0.02 | 0.13 | n.a. |  |  |
| Hospital out-patient (last 12 months) | 0.790 | 0.01 | 0.40 | 0.430 | 0.02 | 0.27 | n.a. |  |  |
| Reported to have seen a doctor (last 12 months) | 0.800 | 0.00 | 0.02 | 0.410 | 0.01 | 0.12 | n.a. |  |  |

Results for NCDS not weighted. Results for BHPS and UKHLS weighted and standard errors adjust for complex survey design.

^1^ t-test of equality of group means, H_0_= no difference in means.

Source: NCDS Sweep 8, BHPS W18, UKHLS W1.
