# Supplementary material for: Analysis of four studies in a comparative framework reveals: health linkage consent rates on British cohort studies higher than on UK household panel surveys
Source: BMC Med Res Methodol. 2014 Nov 27;14:125. doi: 10.1186/1471-2288-14-125 (PMC4280701; doi:10.1186/1471-2288-14-125)
Supplement: Supplementary file 3 — Additional file 3: Table S3: Logistic regressions on consent to health data linkage considering detailed ethnic categories using the UKHLS. Beta coefficients and marginal effects. Logistic regressions on consent to health data linkage for the UKHLS; includes a detailed self-reported ethnic group classification. (DOCX 24 KB) [file 12874_2014_1141_MOESM3_ESM.docx]

**Table S3 - Logistic regressions on consent to health data linkage considering detailed ethnic categories using the UKHLS. Beta-coefficients and marginal effects**

|  | Short version ethnic indicator | | | | Detailed ethnicity indicator | | | |
| --- | --- | --- | --- | --- | --- | --- | --- | --- |
|  | Coeff. | S.E. | ME | S.E. | Coeff. | S.E. | ME | S.E. |
| Ethnic group – short version (ref=British/Irish White) |  |  |  |  |  |  |  |  |
| *White Irish* | 0.02 | 0.15 | 0.00 | 0.03 |  |  |  |  |
| *Other White* | -0.46*** | 0.10 | -0.09*** | 0.02 |  |  |  |  |
| *Mixed* | -0.14 | 0.11 | -0.03 | 0.02 |  |  |  |  |
| *Indian* | -0.29** | 0.09 | -0.06** | 0.02 |  |  |  |  |
| *Pakistani* | -0.87*** | 0.11 | -0.19*** | 0.03 |  |  |  |  |
| *Bangladeshi* | -0.68*** | 0.14 | -0.14*** | 0.03 |  |  |  |  |
| *Caribbean* | -0.47*** | 0.10 | -0.10*** | 0.02 |  |  |  |  |
| *African* | -0.50*** | 0.10 | -0.10*** | 0.02 |  |  |  |  |
| *Other Ethnic Group* | -0.60*** | 0.10 | -0.13*** | 0.02 |  |  |  |  |
| Ethnic group – long version (ref=British/Irish White) |  |  |  |  |  |  |  |  |
| *White Irish* |  |  |  |  | 0.02 | 0.15 | 0.00 | 0.03 |
| *Other White* |  |  |  |  | -0.45*** | 0.10 | -0.09*** | 0.02 |
| *White and Black Caribbean* |  |  |  |  | -0.36* | 0.18 | -0.07 | 0.04 |
| *White and Black African* |  |  |  |  | -0.15 | 0.27 | -0.03 | 0.05 |
| *White and Asian* |  |  |  |  | -0.04 | 0.22 | -0.01 | 0.04 |
| *Other Mixed* |  |  |  |  | 0.06 | 0.23 | 0.01 | 0.04 |
| *Indian* |  |  |  |  | -0.29** | 0.09 | -0.06** | 0.02 |
| *Pakistani* |  |  |  |  | -0.87*** | 0.11 | -0.19*** | 0.03 |
| *Bangladeshi* |  |  |  |  | -0.68*** | 0.14 | -0.14*** | 0.03 |
| *Other Asian* |  |  |  |  | -0.56*** | 0.15 | -0.12*** | 0.04 |
| *Caribbean* |  |  |  |  | -0.47*** | 0.10 | -0.10*** | 0.02 |
| *African* |  |  |  |  | -0.50*** | 0.10 | -0.10*** | 0.02 |
| *Other Black* |  |  |  |  | -0.66* | 0.31 | -0.14 | 0.07 |
| *Chinese* |  |  |  |  | -0.66** | 0.22 | -0.14** | 0.05 |
| *Other Ethnic Group* |  |  |  |  | -0.59*** | 0.15 | -0.12*** | 0.03 |
| England | -0.10 | 0.07 | -0.02 | 0.01 | -0.10 | 0.07 | -0.02 | 0.01 |
| London/SE | -0.10* | 0.05 | -0.02* | 0.01 | -0.10* | 0.05 | -0.02* | 0.01 |
| Male | 0.09*** | 0.03 | 0.02*** | 0.00 | 0.09*** | 0.03 | 0.02*** | 0.00 |
| Aged 50-52 | 0.10 | 0.06 | 0.02 | 0.01 | 0.10 | 0.06 | 0.02 | 0.01 |
| Number of own children in the household (ref: none) |  |  |  |  |  |  |  |  |
| *1* | 0.12* | 0.05 | 0.02* | 0.01 | 0.12* | 0.05 | 0.02* | 0.01 |
| *2* | 0.08 | 0.05 | 0.02 | 0.01 | 0.08 | 0.05 | 0.01 | 0.01 |
| *3 or more* | 0.08 | 0.08 | 0.01 | 0.01 | 0.08 | 0.08 | 0.01 | 0.01 |
| Lives alone | -0.13** | 0.04 | -0.02** | 0.01 | -0.13** | 0.04 | -0.02** | 0.01 |
| Highest degree (ref: higher degree) |  |  |  |  |  |  |  |  |
| *first degree* | 0.06 | 0.06 | 0.01 | 0.01 | 0.06 | 0.06 | 0.01 | 0.01 |
| *Diploma* | 0.16** | 0.06 | 0.03** | 0.01 | 0.16** | 0.06 | 0.03** | 0.01 |
| *A-level* | 0.19** | 0.07 | 0.04** | 0.01 | 0.19** | 0.07 | 0.04** | 0.01 |
| *Other qualification* | 0.15** | 0.05 | 0.03** | 0.01 | 0.15** | 0.05 | 0.03** | 0.01 |
| *No educational qualification* | -0.02 | 0.06 | -0.00 | 0.01 | -0.02 | 0.06 | -0.00 | 0.01 |
| Unemployed | 0.11 | 0.06 | 0.02 | 0.01 | 0.11 | 0.06 | 0.02 | 0.01 |
| Socio-economic status (ref=managerial/professional) |  |  |  |  |  |  |  |  |
| *Intermediate* | -0.04 | 0.06 | -0.01 | 0.01 | -0.04 | 0.06 | -0.01 | 0.01 |
| *Employers* | -0.11 | 0.09 | -0.02 | 0.02 | -0.11 | 0.09 | -0.02 | 0.02 |
| *Supervisory* | 0.09 | 0.07 | 0.02 | 0.01 | 0.08 | 0.07 | 0.02 | 0.01 |
| *Routine* | 0.12* | 0.05 | 0.02* | 0.01 | 0.12* | 0.05 | 0.02* | 0.01 |
| *other status* | -0.08 | 0.07 | -0.02 | 0.01 | -0.08 | 0.07 | -0.02 | 0.01 |
| *Monthly gross earnings (ref: bottom quartile)* |  |  |  |  |  |  |  |  |
| *2nd quartile* | 0.00 | 0.04 | 0.00 | 0.01 | 0.00 | 0.04 | 0.00 | 0.01 |
| *3rd quartile* | -0.15* | 0.08 | -0.03* | 0.01 | -0.15* | 0.07 | -0.03* | 0.01 |
| *4th quartile* | -0.16* | 0.08 | -0.03* | 0.01 | -0.17* | 0.08 | -0.03* | 0.01 |
| Subjective health (ref: excellent) |  |  |  |  |  |  |  |  |
| *Good* | 0.02 | 0.04 | 0.00 | 0.01 | 0.02 | 0.04 | 0.00 | 0.01 |
| *Fair* | -0.02 | 0.05 | -0.00 | 0.01 | -0.02 | 0.05 | -0.00 | 0.01 |
| *Poor* | -0.06 | 0.06 | -0.01 | 0.01 | -0.06 | 0.06 | -0.01 | 0.01 |
| *very poor* | -0.02 | 0.07 | -0.00 | 0.01 | -0.02 | 0.07 | -0.00 | 0.01 |
| Body Mass Index (ref: bottom quartile) |  |  |  |  |  |  |  |  |
| *2nd quartile* | 0.05 | 0.09 | 0.01 | 0.02 | 0.05 | 0.09 | 0.01 | 0.02 |
| *3rd quartile* | 0.00 | 0.09 | 0.00 | 0.02 | 0.00 | 0.09 | 0.00 | 0.02 |
| *4th quartile* | 0.12 | 0.10 | 0.02 | 0.02 | 0.12 | 0.10 | 0.02 | 0.02 |
| Health limits daily activities | 0.06 | 0.04 | 0.01 | 0.01 | 0.06 | 0.04 | 0.01 | 0.01 |
| Suffering from an illness | 0.12* | 0.05 | 0.02* | 0.01 | 0.12* | 0.05 | 0.02* | 0.01 |
| Reported health problem |  |  |  |  |  |  |  |  |
| *Diabetes* | 0.06 | 0.07 | 0.01 | 0.01 | 0.06 | 0.07 | 0.01 | 0.01 |
| *relating to stomach problems* | -0.03 | 0.07 | -0.01 | 0.01 | -0.04 | 0.07 | -0.01 | 0.01 |
| *Cancer* | -0.09 | 0.13 | -0.02 | 0.02 | -0.09 | 0.13 | -0.02 | 0.02 |
| *Epilepsy* | 0.03 | 0.17 | 0.01 | 0.03 | 0.03 | 0.17 | 0.01 | 0.03 |
| *relating to chest problems* | 0.01 | 0.05 | 0.00 | 0.01 | 0.01 | 0.05 | 0.00 | 0.01 |
| *other health problem* | -0.02 | 0.04 | -0.00 | 0.01 | -0.02 | 0.04 | -0.00 | 0.01 |
| Constant | 1.14*** | 0.13 |  |  | 1.13*** | 0.13 |  |  |
| Number of observations | 35,523 |  | 35,523 |  | 35,523 |  | 35,523 |  |

Significant at *** 99%, ** 95%, * 90%.

Results for NCDS not weighted. Results for BHPS and UKHLS weighted and standard errors adjusted for complex survey design.

Source: NCDS Sweep 8, BHPS W18, UKHLS W1.
