# Supplementary material for: Analysis of four studies in a comparative framework reveals: health linkage consent rates on British cohort studies higher than on UK household panel surveys
Source: BMC Med Res Methodol. 2014 Nov 27;14:125. doi: 10.1186/1471-2288-14-125 (PMC4280701; doi:10.1186/1471-2288-14-125)
Supplement: Supplementary file 4 — Additional file 4: Table S4: Logistic regressions on consent to health data linkage on UKHLS sample born in the UK and aged 48–52 (N = 3,144). Beta coefficients and marginal effects. Logistic regressions on consent to health data linkage for the UKHLS; focusing on a subsample of the population that is similar in age to the population studied in the NCDS. (DOCX 18 KB) [file 12874_2014_1141_MOESM4_ESM.docx]

**Table S4 - Logistic regressions on consent to health data linkage on UKHLS sample born in the UK and aged 48-52 (N=3,144). Beta-coefficients and marginal effects**

|  | Coeff. | S.E. | ME | S.E. |
| --- | --- | --- | --- | --- |
| England | 0.00 | 0.17 | 0.00 | 0.03 |
| London/SE | -0.12 | 0.13 | -0.02 | 0.02 |
| Male | 0.12 | 0.10 | 0.02 | 0.02 |
| British/Irish White | 0.29 | 0.26 | 0.05 | 0.05 |
| Aged 50-52 | 0.16 | 0.10 | 0.03 | 0.02 |
| Number of own children in the household (ref: none) |  |  |  |  |
| *1* | 0.34* | 0.15 | 0.06* | 0.03 |
| *2* | 0.02 | 0.20 | 0.00 | 0.04 |
| *3 or more* | 0.56 | 0.41 | 0.11 | 0.08 |
| Lives alone | -0.18 | 0.15 | -0.03 | 0.03 |
| Highest degree (ref: higher degree) |  |  |  |  |
| *first degree* | -0.29 | 0.21 | -0.05 | 0.04 |
| *Diploma* | -0.12 | 0.21 | -0.02 | 0.04 |
| *A-level* | -0.22 | 0.26 | -0.04 | 0.05 |
| *Other qualification* | 0.02 | 0.19 | 0.00 | 0.04 |
| *No educational qualification* | -0.17 | 0.24 | -0.03 | 0.05 |
| Unemployed | -0.16 | 0.23 | -0.03 | 0.04 |
| Socio-economic status (ref=managerial/professional) |  |  |  |  |
| *Intermediate* | 0.21 | 0.18 | 0.04 | 0.03 |
| *Employers* | -0.26 | 0.33 | -0.05 | 0.06 |
| *Supervisory* | 0.23 | 0.23 | 0.04 | 0.04 |
| *Routine* | 0.02 | 0.17 | 0.00 | 0.03 |
| *other status* | -0.33 | 0.32 | -0.06 | 0.06 |
| *Monthly gross earnings (ref: bottom quartile)* |  |  |  |  |
| *2nd quartile* | 0.04 | 0.18 | 0.01 | 0.03 |
| *3rd quartile* | -0.39 | 0.31 | -0.07 | 0.06 |
| *4th quartile* | -0.35 | 0.30 | -0.07 | 0.06 |
| Subjective health (ref: excellent) |  |  |  |  |
| *Good* | -0.09 | 0.15 | -0.02 | 0.03 |
| *Fair* | -0.20 | 0.16 | -0.04 | 0.03 |
| *Poor* | -0.23 | 0.19 | -0.04 | 0.04 |
| *very poor* | -0.47 | 0.28 | -0.09 | 0.05 |
| Body Mass Index (ref: bottom quartile) |  |  |  |  |
| *2nd quartile* | -0.33 | 0.52 | -0.06 | 0.10 |
| *3rd quartile* | -0.40 | 0.52 | -0.07 | 0.10 |
| *4th quartile* | -0.28 | 0.52 | -0.05 | 0.10 |
| Health limits daily activities | 0.04 | 0.14 | 0.01 | 0.03 |
| Suffering from an illness | 0.07 | 0.17 | 0.01 | 0.03 |
| Reported health problem |  |  |  |  |
| *Diabetes* | 0.25 | 0.29 | 0.05 | 0.05 |
| *relating to stomach problems* | -0.04 | 0.25 | -0.01 | 0.05 |
| *Cancer* | -0.03 | 0.45 | -0.01 | 0.09 |
| *Epilepsy* | -0.05 | 0.49 | -0.01 | 0.09 |
| *relating to chest problems* | 0.10 | 0.18 | 0.02 | 0.03 |
| *other health problem* | 0.21 | 0.14 | 0.04 | 0.03 |
| Constant | 1.50* | 0.68 |  |  |
| Number of observations | 26,910 |  | 26,910 |  |

Significant at *** 99%, ** 95%, * 90%.

Results for NCDS not weighted. Results for BHPS and UKHLS weighted and standard errors adjusted for complex survey design.

Source: NCDS Sweep 8, BHPS W18, UKHLS W1
