# Supplementary material for: Analysis of four studies in a comparative framework reveals: health linkage consent rates on British cohort studies higher than on UK household panel surveys
Source: BMC Med Res Methodol. 2014 Nov 27;14:125. doi: 10.1186/1471-2288-14-125 (PMC4280701; doi:10.1186/1471-2288-14-125)
Supplement: Supplementary file 6 — Additional file 6: Table S6: Logistic regressions on consent to health data linkage on five random samples (RS) of the UKHLS. Marginal effects. Logistic regressions on consent to health data linkage for the UKHLS; focusing on five random subsamples of the population so that the sample size is more similar to that studied in the NCDS and the BHPS. Results reported as marginal effects. (DOCX 22 KB) [file 12874_2014_1141_MOESM6_ESM.docx]

**Table S6 - Logistic regressions on consent to health data linkage on five random samples (RS) of the UKHLS. Marginal effects.**

|  | RS1 | | RS2 | | RS3 | | RS4 | | RS5 | |
| --- | --- | --- | --- | --- | --- | --- | --- | --- | --- | --- |
|  | ME | S.E. | ME | S.E. | ME . | S.E. | ME | S.E. | ME | S.E. |
| England | -0.03 | 0.02 | -0.01 | 0.02 | 0.00 | 0.02 | -0.04 | 0.02 | -0.02 | 0.02 |
| London/SE | -0.01 | 0.02 | -0.00 | 0.02 | -0.03* | 0.02 | -0.03* | 0.02 | -0.02 | 0.02 |
| Male | 0.03* | 0.01 | 0.01 | 0.01 | 0.02 | 0.01 | 0.01 | 0.01 | 0.00 | 0.01 |
| British/Irish White | 0.06*** | 0.02 | 0.09*** | 0.02 | 0.11*** | 0.02 | 0.08*** | 0.02 | 0.11*** | 0.02 |
| Aged 50-52 | -0.02 | 0.03 | 0.05 | 0.03 | 0.02 | 0.03 | 0.03 | 0.03 | 0.03 | 0.03 |
| Number of own children in the household (ref: none) |  |  |  |  |  |  |  |  |  |  |
| *1* | 0.03 | 0.02 | 0.04 | 0.02 | 0.02 | 0.02 | 0.01 | 0.02 | 0.02 | 0.02 |
| *2* | 0.03 | 0.02 | -0.00 | 0.02 | 0.01 | 0.02 | 0.02 | 0.02 | 0.01 | 0.02 |
| *3 or more* | -0.00 | 0.03 | 0.02 | 0.03 | -0.01 | 0.03 | 0.04 | 0.03 | 0.00 | 0.03 |
| Lives alone | -0.03* | 0.02 | -0.01 | 0.02 | -0.00 | 0.02 | -0.02 | 0.02 | -0.06** | 0.02 |
| Highest degree (ref: higher degree) |  |  |  |  |  |  |  |  |  |  |
| *first degree* | -0.00 | 0.02 | 0.00 | 0.02 | -0.00 | 0.02 | 0.01 | 0.02 | 0.04 | 0.02 |
| *Diploma* | 0.04 | 0.02 | 0.01 | 0.03 | 0.01 | 0.02 | 0.03 | 0.03 | 0.05* | 0.03 |
| *A-level* | 0.06* | 0.03 | 0.02 | 0.03 | 0.02 | 0.03 | 0.05 | 0.03 | 0.04 | 0.03 |
| *Other qualification* | 0.06** | 0.02 | 0.01 | 0.02 | 0.01 | 0.02 | 0.03 | 0.02 | 0.03 | 0.02 |
| *No educational qualification* | 0.00 | 0.02 | -0.03 | 0.03 | -0.01 | 0.02 | -0.02 | 0.02 | 0.03 | 0.02 |
| Unemployed | 0.00 | 0.03 | 0.03 | 0.03 | 0.03 | 0.03 | -0.01 | 0.03 | 0.05 | 0.03 |
| Socio-economic status (ref=managerial/professional) |  |  |  |  |  |  |  |  |  |  |
| *Intermediate* | 0.01 | 0.02 | 0.01 | 0.02 | -0.02 | 0.03 | -0.05 | 0.03 | -0.00 | 0.03 |
| *Employers* | 0.04 | 0.04 | 0.07 | 0.04 | -0.06 | 0.04 | -0.10* | 0.04 | -0.04 | 0.04 |
| *Supervisory* | -0.01 | 0.03 | 0.05 | 0.03 | 0.01 | 0.04 | -0.01 | 0.03 | 0.04 | 0.03 |
| *Routine* | 0.02 | 0.02 | 0.03 | 0.02 | 0.03 | 0.02 | -0.02 | 0.02 | 0.03 | 0.02 |
| *other status* | 0.01 | 0.03 | 0.04 | 0.03 | -0.04 | 0.03 | -0.03 | 0.03 | -0.05 | 0.04 |
| *Monthly gross earnings (ref: bottom quartile)* |  |  |  |  |  |  |  |  |  |  |
| *2nd quartile* | 0.03 | 0.02 | -0.02 | 0.02 | 0.00 | 0.02 | 0.01 | 0.02 | -0.01 | 0.02 |
| *3rd quartile* | -0.02 | 0.03 | -0.02 | 0.03 | -0.02 | 0.03 | -0.01 | 0.03 | -0.07 | 0.04 |
| *4th quartile* | 0.02 | 0.03 | -0.02 | 0.03 | -0.04 | 0.03 | -0.05 | 0.03 | -0.06 | 0.04 |
| Subjective health (ref: excellent) |  |  |  |  |  |  |  |  |  |  |
| *Good* | 0.01 | 0.02 | 0.03 | 0.02 | -0.00 | 0.02 | 0.00 | 0.02 | -0.01 | 0.02 |
| *Fair* | 0.01 | 0.02 | 0.01 | 0.02 | -0.02 | 0.02 | -0.02 | 0.02 | -0.00 | 0.02 |
| *Poor* | -0.04 | 0.02 | -0.00 | 0.02 | -0.01 | 0.03 | -0.00 | 0.02 | -0.01 | 0.03 |
| *very poor* | 0.00 | 0.03 | 0.00 | 0.03 | -0.04 | 0.03 | 0.01 | 0.03 | 0.01 | 0.03 |
| Body Mass Index (ref: bottom quartile) |  |  |  |  |  |  |  |  |  |  |
| *2nd quartile* | 0.03 | 0.04 | -0.01 | 0.05 | 0.04 | 0.04 | -0.03 | 0.04 | 0.00 | 0.04 |
| *3rd quartile* | 0.01 | 0.04 | -0.01 | 0.05 | 0.04 | 0.04 | -0.04 | 0.04 | 0.00 | 0.04 |
| *4th quartile* | 0.03 | 0.04 | 0.02 | 0.05 | 0.06 | 0.04 | -0.01 | 0.05 | 0.02 | 0.04 |
| Health limits daily activities | 0.05** | 0.02 | 0.01 | 0.02 | 0.01 | 0.02 | -0.01 | 0.02 | -0.00 | 0.02 |
| Suffering from an illness | 0.00 | 0.02 | 0.03 | 0.02 | 0.05* | 0.02 | 0.00 | 0.02 | 0.02 | 0.02 |
| Reported health problem |  |  |  |  |  |  |  |  |  |  |
| *Diabetes* | 0.05 | 0.03 | -0.02 | 0.03 | 0.02 | 0.03 | 0.02 | 0.03 | 0.00 | 0.03 |
| *relating to stomach problems* | -0.02 | 0.03 | -0.02 | 0.03 | -0.02 | 0.03 | -0.02 | 0.03 | 0.05 | 0.03 |
| *Cancer* | -0.04 | 0.06 | -0.07 | 0.06 | -0.07 | 0.06 | 0.08 | 0.06 | 0.03 | 0.06 |
| *Epilepsy* | 0.06 | 0.07 | 0.01 | 0.08 | -0.06 | 0.07 | 0.11 | 0.08 | -0.08 | 0.07 |
| *relating to chest problems* | 0.02 | 0.02 | -0.02 | 0.02 | 0.00 | 0.02 | 0.00 | 0.02 | 0.01 | 0.02 |
| *other health problem* | 0.01 | 0.02 | -0.03 | 0.02 | -0.01 | 0.02 | 0.01 | 0.02 | 0.00 | 0.02 |
| Number of observations | 7,088 |  | 7,075 |  | 7,093 |  | 7,084 |  | 7,080 |  |

Significant at *** 99%, ** 95%, * 90%.

Results for NCDS not weighted. Results for BHPS and UKHLS weighted and standard errors adjusted for complex survey design.

Source: NCDS Sweep 8, BHPS W18, UKHLS W1.
