# Supplementary material for: Analysis of four studies in a comparative framework reveals: health linkage consent rates on British cohort studies higher than on UK household panel surveys
Source: BMC Med Res Methodol. 2014 Nov 27;14:125. doi: 10.1186/1471-2288-14-125 (PMC4280701; doi:10.1186/1471-2288-14-125)
Supplement: Supplementary file 8 — Additional file 8: Table S8: Logistic regressions on consent to health data linkage including markers of survey co-operation. Marginal effects. Logistic regressions on consent to health data linkage for the NCDS, BHPS and UKHLS studies; includes additional predictor variables to allow for heterogeneity in the study participants’ degree of survey co-operation. Results reported as marginal effects. (DOCX 21 KB) [file 12874_2014_1141_MOESM8_ESM.docx]

**Table S8 - Logistic regressions on consent to health data linkage including markers of survey co-operation. Marginal effects.**

|  | **NCDS** | | **BHPS** | | **UKHLS** | |
| --- | --- | --- | --- | --- | --- | --- |
|  | **M.E.** | **S.E.** | **M.E.** | **S.E.** | **M.E.** | **S.E.** |
| England | 0.01 | 0.01 | 0.07 | 0.04 | -0.01 | 0.01 |
| London/SE | -0.02* | 0.01 | 0.03 | 0.03 | -0.02* | 0.01 |
| Male | 0.01 | 0.01 | 0.03* | 0.01 | 0.02*** | 0.00 |
| British/Irish White | 0.05* | 0.02 | 0.18*** | 0.03 | 0.08*** | 0.01 |
| Aged 50-52 |  |  | 0.01 | 0.03 | 0.02 | 0.01 |
| Number of own children in the household (ref: none) |  |  |  |  |  |  |
| *1* | -0.02* | 0.01 | 0.01 | 0.03 | 0.02* | 0.01 |
| *2* | -0.01 | 0.01 | -0.00 | 0.03 | 0.01 | 0.01 |
| *3 or more* | -0.02 | 0.02 | 0.08 | 0.04 | 0.01 | 0.01 |
| Lives alone | 0.03* | 0.02 | 0.04 | 0.02 | -0.03*** | 0.01 |
| Highest degree (ref: higher degree) |  |  |  |  |  |  |
| *first degree* | -0.02 | 0.03 | -0.07 | 0.05 | 0.01 | 0.01 |
| *diploma* | -0.02 | 0.03 | -0.08 | 0.05 | 0.03** | 0.01 |
| *A-level* | -0.02 | 0.03 | -0.04 | 0.05 | 0.04** | 0.01 |
| *Other qualification* | -0.00 | 0.02 | -0.06 | 0.05 | 0.04*** | 0.01 |
| *No educational qualification* | -0.03 | 0.03 | -0.10* | 0.05 | 0.00 | 0.01 |
| Unemployed | 0.02 | 0.03 | 0.04 | 0.05 | 0.03* | 0.01 |
| Socio-economic status (ref=managerial/professional) |  |  |  |  |  |  |
| *intermediate* | -0.01 | 0.02 | -0.01 | 0.03 | -0.01 | 0.01 |
| *employers* | 0.01 | 0.02 | -0.11* | 0.05 | -0.03 | 0.02 |
| *supervisory* | 0.02 | 0.02 | 0.03 | 0.04 | 0.02 | 0.01 |
| *routine* | 0.02 | 0.01 | 0.01 | 0.03 | 0.02* | 0.01 |
| *other status* | -0.00 | 0.02 | -0.03 | 0.04 | -0.03 | 0.01 |
| Monthly gross earnings (ref: bottom quartile) |  |  |  |  |  |  |
| *2nd quartile* | 0.01 | 0.01 | -0.02 | 0.02 | 0.00 | 0.01 |
| *3rd quartile* | 0.06*** | 0.02 | -0.00 | 0.05 | -0.01 | 0.01 |
| *4th quartile* | 0.06*** | 0.02 | -0.04 | 0.05 | -0.02 | 0.01 |
| Votes for left-wing party | 0.02 | 0.01 | 0.05** | 0.02 | 0.04*** | 0.01 |
| Non-voter | -0.01 | 0.01 | -0.06* | 0.02 | -0.01 | 0.02 |
| Refused income question | -0.26*** | 0.03 | -0.35*** | 0.08 | -0.27*** | 0.01 |
| Generally trusts others | 0.04*** | 0.01 | 0.07*** | 0.02 | 0.04*** | 0.01 |
| Subjective health (ref: excellent) |  |  |  |  |  |  |
| *Good* | 0.00 | 0.01 | -0.03 | 0.02 | 0.00 | 0.01 |
| *Fair* | -0.04** | 0.01 | -0.06* | 0.03 | 0.00 | 0.01 |
| *Poor* | 0.01 | 0.02 | -0.03 | 0.04 | -0.01 | 0.01 |
| *very poor* | 0.02 | 0.03 | 0.04 | 0.06 | 0.00 | 0.01 |
| Body Mass Index (ref: bottom quartile) |  |  |  |  |  |  |
| *2nd quartile* | 0.08* | 0.04 | -0.05 | 0.05 | 0.01 | 0.02 |
| *3rd quartile* | 0.09* | 0.04 | -0.04 | 0.05 | -0.00 | 0.02 |
| *4th quartile* | 0.12** | 0.04 | 0.02 | 0.05 | 0.02 | 0.02 |
| Health limits daily activities | -0.01 | 0.02 | 0.00 | 0.03 | 0.01 | 0.01 |
| Suffering from an illness | 0.02 | 0.02 | 0.05 | 0.04 | 0.02* | 0.01 |
| Reported health problem |  |  |  |  |  |  |
| *Diabetes* | -0.03 | 0.02 | 0.04 | 0.03 | 0.01 | 0.01 |
| *relating to stomach problems* | 0.04* | 0.02 | 0.02 | 0.03 | -0.01 | 0.01 |
| *Cancer* | 0.02 | 0.05 | 0.09 | 0.06 | -0.02 | 0.02 |
| *Epilepsy* | -0.02 | 0.05 | -0.00 | 0.08 | 0.01 | 0.03 |
| *relating to chest problems* | 0.03* | 0.01 | -0.00 | 0.02 | 0.00 | 0.01 |
| *other health problem* | 0.00 | 0.02 | -0.01 | 0.03 | -0.01 | 0.01 |
| Number of observations | 9,264 |  | 5,881 |  | 35,536 |  |

Significant at *** 99%, ** 95%, * 90%.

Results for NCDS not weighted. Results for BHPS and UKHLS weighted and standard errors adjusted for complex survey design.

Source: NCDS Sweep 8, BHPS W18, UKHLS W1.
